# A complete mitochondrial genome of the large scaled tongue sole (*cynoglossus macrolepidotus*) from the east China sea

**DOI:** 10.1080/23802359.2025.2509016

**Published:** 2025-05-19

**Authors:** Haojie Zhuo, Hao Yuan, Guang Yang

**Affiliations:** College of Life Science, Nanjing Normal University, Nanjing, China

**Keywords:** Mithogenome, cynoglossus, phylogenetics

## Abstract

The Large-scaled tongue sole (*Cynoglossus macrolepidotus*, Bleeker, 1851) is an economically important fish species in China. This study presents the first complete mitochondrial genome of the Large-scaled tongue sole, with a total length of 16,647 base pairs. The genome includes 13 protein-coding genes (PCGs), 22 transfer RNA (tRNA) genes, and two ribosomal RNA (rRNA) genes. Phylogenetic analysis reveals that *C. macrolepidotus* is genetically closer to *Cynoglossus joyneri* and *Cynoglossus puncticeps* than to other species within the *Cynoglossus* genus. This study provides valuable genetic data for future research on *C. macrolepidotus* and contributes to phylogenetic studies within the Cynoglossidae family.

## 1. Introduction

The Large scaled tongue sole belongs to the Cynoglossidae family and has a flattened, tongue-like body. It has a short head and small eyes located on the left side. Its body is light brown with two lateral lines, and the dorsal and anal fins are fused with the tail fin (Su et al. 2011). This species is distributed from the Persian Gulf to Indonesia and the Philippines, including Hainan Island, Guangdong, Guangxi, Fujian, and Taiwan. The type locality is the East Indies. Large scaled tongue sole is a benthic marine fish that lives in warm waters. It inhabits muddy bottom areas nearshore and feeds on benthic invertebrates (Sharma and Jaiswar 2023). It can also be found in estuarine or tidal river areas.

The investigation of the complete mitochondrial genome of Large scaled tongue sole is of considerable significance due to its substantial economic advantages. This species has emerged as an important aquaculture species in China because of its low bone content, superior taste, and high nutritional profile (Bureau MOAA 2022). This study presents the complete mitochondrial genome of Large scaled tongue sole and its phylogenetic analysis. The results provide valuable genetic resources for future research and species identification.

## 2. Materials and methods

**Figure 1.**
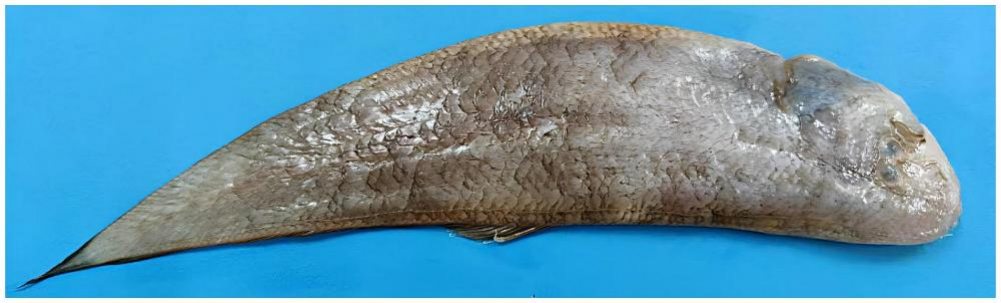
A reference image of the large scaled tongue sole sequenced in this work, collected by Haojie Zhuo in Xiamen (24.5450000°N, 118.3808333°E), China. Photographed by Haojie Zhuo at the laboratory of life science college, Nanjing Normal University.

In October 2023, a specimen of Large scaled tongue sole was collected through bottom trawling by Haojie Zhuo in Xiamen, Fujian Province, China (Latitude: 118.3808333, Longitude: 24.5450000) (Figure 1). This Large scaled tongue sole was still alive when captured and was placed in a freezer at minus twenty degrees on the same day, and it was photographed after eight months. The specimen was identified based on its morphological characteristics. It was subsequently sent to Nanjing and preserved at −40 °C in Nanjing Normal University (http://sky.njnu.edu.cn/index.htm, Haojie Zhuo: 1830033512@qq.com) with the voucher ID 20231027xiamen059.

Genomic DNA extraction and sequencing followed standard protocols and manufacturer guidelines (Arakawa et al. 2018; Kono et al. 2021). DNA was extracted from the entire organism. The prepared sequencing library was analyzed using the Illumina NovaSeq 6000 platform with 150 bp paired-end reads. Trimmomatic 0.39 was used to filter raw reads before assembly (Bolger et al. 2014). The mitochondrial genome was assembled using GetOrganelle v1.7.5 (Jin et al. 2020). This tool retrieves target reads iteratively with a seed database and then uses SPAdes for assembly. Candidate sequences were selected based on coverage depth and assembly length. These sequences were aligned to a nucleotide (NT) library to validate mitochondrial scaffolds and then connected using overlapping regions. The sequence orientation and starting position were determined with reference genomes. This process generated the complete mitochondrial genome sequence. Protein-coding genes, tRNA, and rRNA were predicted using MITOS2 (Donath et al. 2019). Duplicated predicted genes were removed, and start/stop codons were manually corrected for accuracy. CGView (Stothard et al. 2019) was used to create a circular map of the genome. Protein sequences were annotated by comparing them to databases such as NCBI non-redundant (Nr), Swiss-Prot, COGs, KEGG, and GO. Blastp was used with an e-value threshold of <1e-5. Only the best alignment for each gene was retained for functional annotation to ensure biological relevance.

A phylogenetic tree was constructed using maximum likelihood (ML) methods. Mitochondrial genome sequences from *Cynoglossus* and *Soleidae* species were retrieved from NCBI. Amino acid sequences were aligned with MACSEv2.07 (Ranwez et al. 2011). Alignment trimming was performed with FasParser v2.13.0 (Sun 2017). Phylogenetic analysis was conducted with the IQ-TREE web server (http://iqtree.cibiv.univie.ac.at/). *Solea senegalensis* and *Zebrias zebra* were used as outgroup taxa. The tree was visualized with FigTree v1.4.4 (http://tree.bio.ed.ac.uk/software/figtree/). The dataset included 15 species: *Cynoglossus roulei, C. semilaevis, C. abbreviatus, C. gracilis, C. puncticeps, C. joyneri, C. macrolepidotus, C. itinus, C. nanhaiensis, C. bilineatus, C. sinicus, C. senegalensis, C. zanzibarensis, Solea senegalensis* and *Zebrias zebra*.

## 3. Results

**Figure 2.**
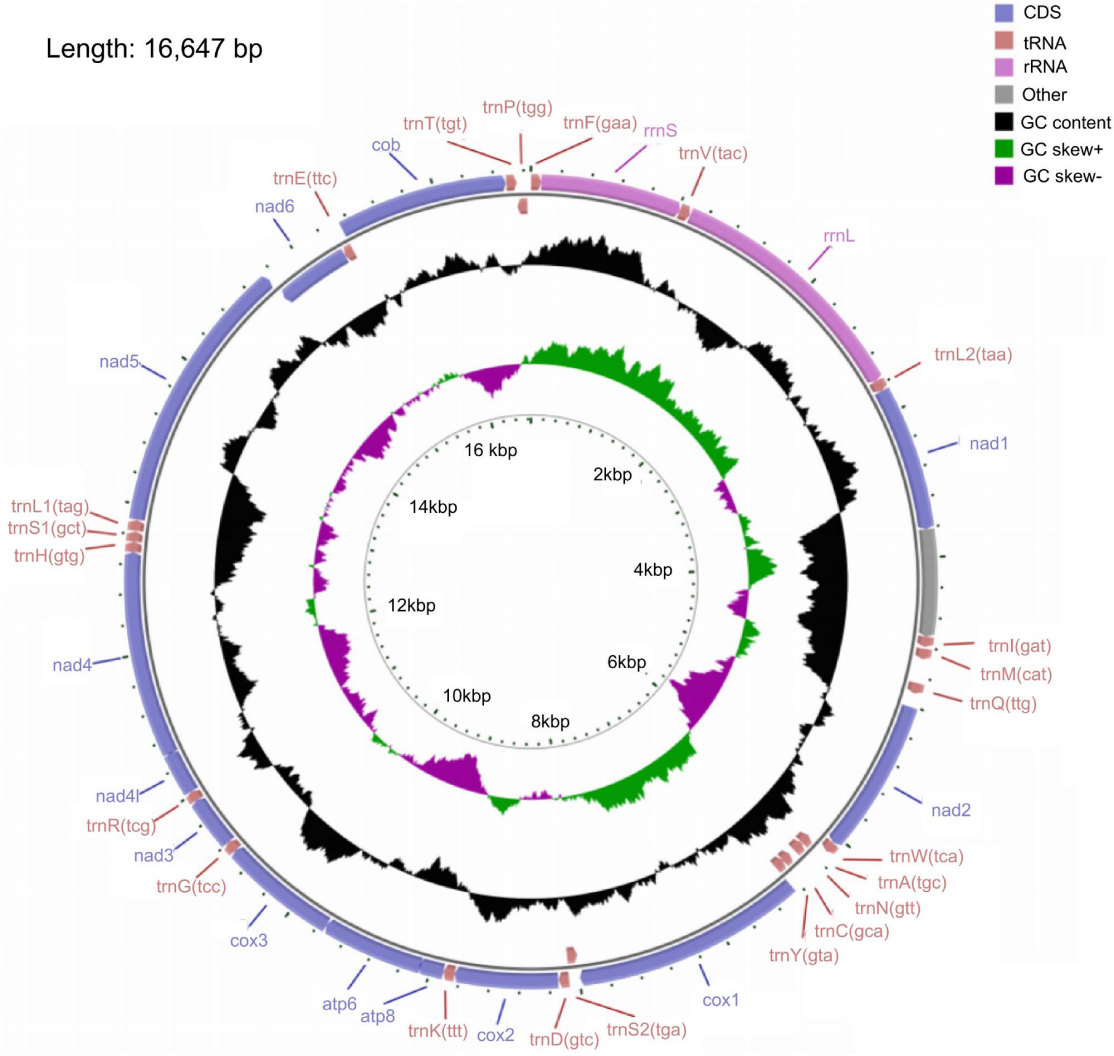
Circular mitogenome map of large scaled tongue sole. The genes scattered on the heavy strand are shown on the outer side of the circle, while the inner side shows those that are scattered on the light strand.

This study provides a comprehensive characterization of the complete mitochondrial genome of Large scaled tongue sole, which spans 16,647 base pairs (bp) and encompasses a total of 37 genes, including 13 protein-coding genes (PCGs), 22 transfer RNA (tRNA) genes, and 2 ribosomal RNA (rRNA) genes (Figure 2). The cumulative length of the PCGs is 11,420 bp, while the tRNA genes account for 1,548 bp, and the rRNA genes total 2,625 bp. Notably, the large ribosomal RNA (lrRNA) measures 1,672 bp, and the small ribosomal RNA (srRNA) is 950 bp in length. The overall base composition of this mitochondrial genome is characterized by 30.59% adenine (A), 24.53% cytosine (C), 30.02% thymine (T), and 14.85% guanine (G), resulting in a guanine-cytosine (GC) content of 39.38%. The coding genes exhibit a pronounced enrichment of adenine and thymine, with an AT content of 60.39%. Most genes are situated on the heavy (H) strand, with the exceptions of trnA (tgc), trnN (gtt), trnC (gca), trnY (gta), trnS2 (tga), nad6, trnE (ttc), and trnP (tgg). All tRNA genes are capable of adopting a typical cloverleaf structure, with lengths ranging from 66 to 75 bp. The coverage-depth map can be found in Supplementary table S1.

**Figure 3.**
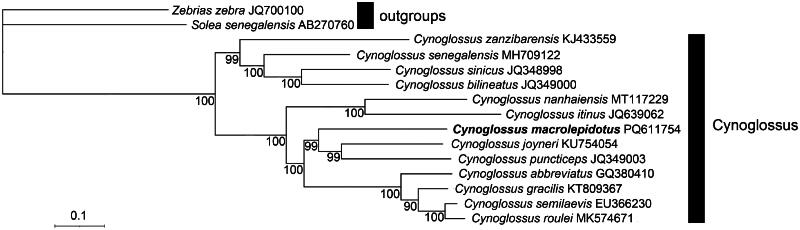
Phylogenetic tree showing the relationship between large scaled tongue sole and other 14 species. The following mitogenome sequences were used: *C. roulei* (MK574671)(Chen et al. 2019), *C. semilaevis* (EU366230)(Kong et al. 2009), *C. abbreviatus* (GQ380410)(Shi et al. 2016), *C. gracilis* (KT809367)(Wei et al. 2016), *C. joyneri* (KU754054)(unpublished), *C. macrolepidotus* (PQ611754), *C. itinus* (JQ639062) (unpublished), *C. nanhaiensis* (MT 117229)(Tian et al. 2020), *C. bilineatus* (JQ349000) (unpublished), *C. sinicus* (JQ348998) (unpublished), *C. senegalensis* (MH709122)(Zealous et al. 2018), *C. zanzibarensis* (KJ433559) (Shi et al. 2016), *S. senegalensis* (AB270760)(Manchado et al. 2007), *Z. zebra* (JQ700100)(Wang et al. 2013). *S. senegalensis* and *Z. zebra* functioned as outgroup.

Phylogenetic analysis utilizing maximum likelihood (ML) methods reveals that Large scaled tongue sole shares a closer genetic relationship with *C. joyneri* and *C. puncticeps* compared to other species within the *Cynoglossus* genus (Figure 3). The molecular phylogenetic insights derived from mitochondrial genomic data enhance our understanding of traditional morphological classifications.

## 4. Discussion and conclusions

Previously, individual mitochondrial gene sequences of the Large-scaled tongue sole have been documented in GenBank. However, the complete mitochondrial genome has not been fully characterized. This study provides the first detailed analysis of the species’ complete mitochondrial genome. The genome spans 16,647 base pairs and contains 37 genes. These include 13 protein-coding genes (PCGs), 22 transfer RNAs (tRNAs), and 2 ribosomal RNAs (rRNAs). Phylogenetic analysis indicates that the Large-scaled tongue sole exhibits a closer genetic relationship to *C. joyneri* and *C. puncticeps* than to other species within the Cynoglossus genus. Furthermore, the interrelationships among the remaining species align with findings from prior phylogenetic studies (Wang et al. 2013; Campbell et al. 2014; Hu et al. 2023). The analyses also provide consistent evidence supporting the monophyly of the Cynoglossidae family and its clear distinction from the Soleidae family (solefishes) within the order Pleuronectiformes (Wang et al. 2020). However, these findings do not support the hypotheses put forth by Liu et al. (2010) concerning the potential synonymy of *C. joyneri* and *C. abbreviatus*, nor do they validate the contentious species status of *C. semilaevis* and *C. gracilis*. The mitochondrial genomic data obtained in this study serve as a valuable genetic resource for future research on the Large-scaled tongue sole. This information lays a foundation for phylogenetic studies within the Cynoglossidae family. It is expected to support hybridization identification, biodiversity monitoring, conservation efforts, DNA barcoding, and population genetics research.

## Supplementary Material

Supplementary Table Sl.tif

## Data Availability

The consensus genome sequence is openly available in GenBank of NCBI at [https://www.ncbi.nlm.nih.gov] under the accession no. PQ611754. The associated BioProject, SRA, and Bio-Sample numbers are PRJNA1184785, SRR31307733, and SAMN44675529 respectively.
