# Supplementary figures and images for: A complete mitochondrial genome of the large scaled tongue sole (cynoglossus macrolepidotus) from the east China sea
Source: Mitochondrial DNA B Resour. 2025 May 19;10(6):518–21. doi: 10.1080/23802359.2025.2509016 (PMC12090253; doi:10.1080/23802359.2025.2509016)

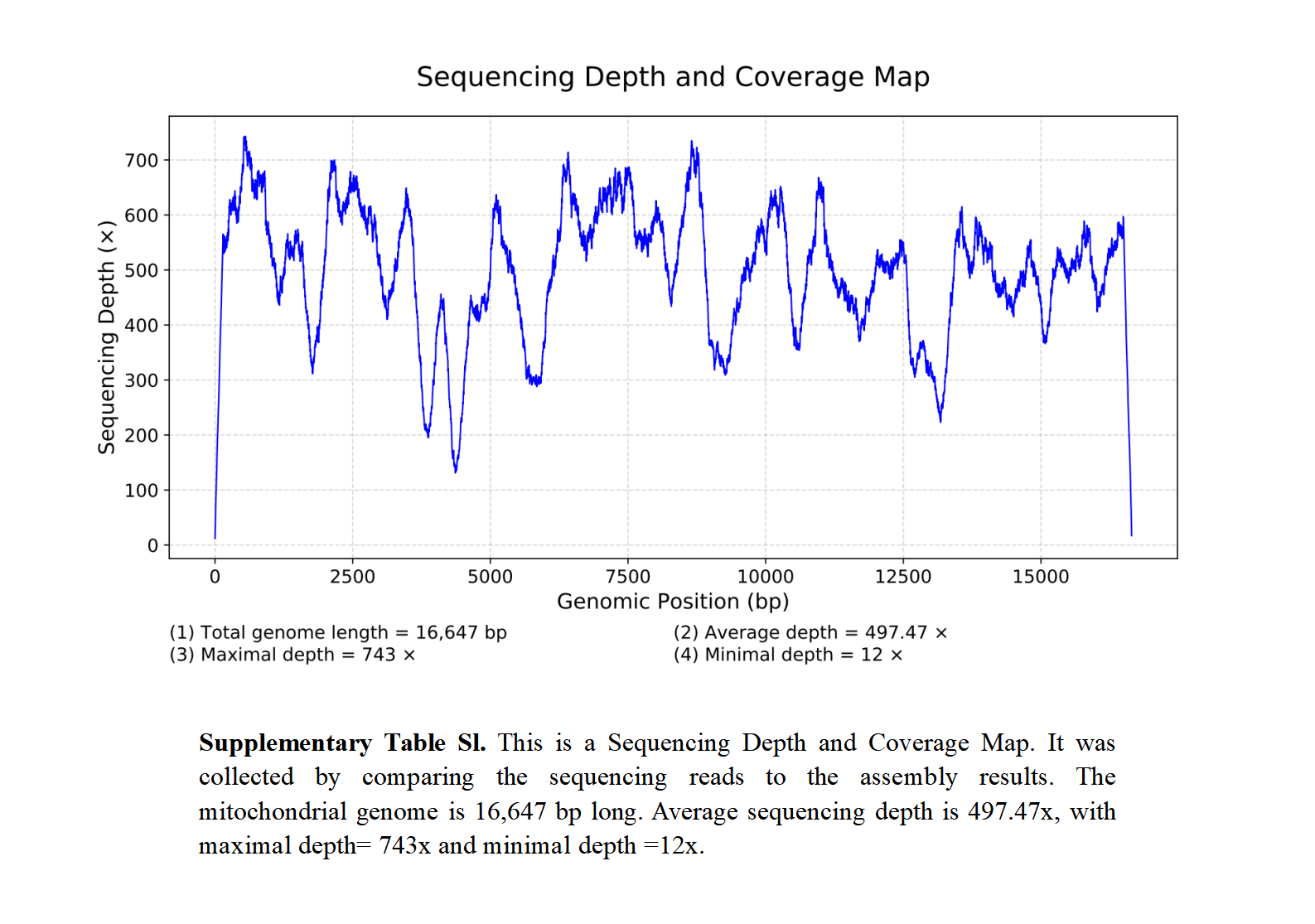

Supplement: Supplementary Table Sl.tif [file TMDN_A_2509016_SM6054.tif]
